# (E)-4-(3,4-Dimethoxyphenyl)but-3-en-1-ol Enhances Melanogenesis through Increasing Upstream Stimulating Factor-1-Mediated Tyrosinase Expression

**DOI:** 10.1371/journal.pone.0141988

**Published:** 2015-11-04

**Authors:** Jisu Park, Heesung Chung, Seung Hyun Bang, Ah-Reum Han, Eun-Kyoung Seo, Sung Eun Chang, Duk-Hee Kang, Eok-Soo Oh

**Affiliations:** 1 Department of Life Sciences, the Research Center for Cellular Homeostasis, Ewha Womans University, Seoul, Korea; 2 Department of Dermatology, University of Ulsan College of Medicine, Asan Medical Center, Seoul, Korea; 3 The Global Top5 Research Program, College of Pharmacy, Ewha Womans University, Seoul, Korea; 4 Division of Nephrology, Department of Internal Medicine, Ewha Medical Research Center, Ewha Womans University School of Medicine, Seoul, Korea; Virginia Commonwealth University, UNITED STATES

## Abstract

We investigated the potential melanogenic effect of compounds from *Zingiber cassumunar* Roxb. Our data revealed that chloroform-soluble extract of *Z*. *cassumunar* enhanced melanin synthesis in B16F10 melanoma cells. Among the components of the chloroform extract, (E)-4-(3,4-dimethoxyphenyl)but-3-en-1-ol (DMPB) increased melanogenesis in both B16F10 cells and human primary melanocytes. In B16F10 cells, DMPB enhanced the activation of ERK and p38, and the level of tyrosinase. Although the level of microphthalmia-associated transcription factor was unchanged in DMPB-treated B16F10 cells, DMPB increased levels and nuclear localization of upstream stimulating factor-1 (USF1). Consistently, DMPB-mediated melanin synthesis was diminished in USF1-knockdown cells. Furthermore, DMPB induced hyperpigmentation in brown guinea pigs *in vivo*. Together, these data suggest that DMPB may promote melanin synthesis via USF1 dependent fashion and could be used as a clinical therapeutic agent against hypopigmentation-associated diseases.

## Introduction

Melanin, which is synthesized in the melanosomes of melanocytes, serves a number of valuable functions, such as determining the appearance of the skin and protecting it from the harmful effects of ultra violet (UV) radiation (and thus skin cancer), toxic drugs and chemicals [1]. Excessive melanin production occurs in melasma, lentigo, nevocellular nevi and malignant melanoma, whereas the loss of melanocyte function leads to vitiligo. Therefore, it is critical to appropriately control the balance of melanin synthesis in the skin. Melanin synthesis is controlled by various enzymes, such as tyrosinase, tyrosinase-related protein 1 (TRP-1) and tyrosinase-related protein 2 (TRP-2/DOPA, chrome tautomerase). Tyrosinase is a rate-limiting enzyme for melanin synthesis, where it is involved in two distinct reactions: it first catalyzes the hydroxylation of tyrosine to 3,4-dihydroxyphenylalanine (DOPA), and then promotes the oxidation of DOPA to DOPA quinone [2], which undergoes several reactions to eventually form melanin. Therefore, many researchers have sought to control the expression or activation of tyrosinase. The transcription of melanogenic enzymes is regulated by microphthalmia-associated transcription factor (MITF) [3] and tyrosinase transcription is also regulated by USF (upstream stimulating factor)-1, which is member of the evolutionarily conserved basic-helix-loop-helix family of eukaryotic leucine zipper transcription factors [4]. In the tyrosinase promoter, the elements recognized by MITF are also targeted by USF1 [5]. In addition, an essential role of p53, a tumor suppressor protein, in the induction of UV-induced epidermal hyperpigmentation via direct activation of *POMC* transcription in keratinocytes [6] and/or regulation of paracrine cytokine signaling, both in keratinocytes and melanocytes, has been reported [7].

Numerous studies have sought to identify the factors involved in controlling melanin synthesis. A number of natural products have been reported to inhibit melanogenesis by regulating melanogenic enzymes, including Hoelen extracts [8], sesamol (3,4-methylenedioxyphenol) [9]. In addition, *Arthrophytum scoparium* extract [10], Caffeoylserotonin [11] and the aqueous fraction from *Cuscuta japonica* [12] have been shown to inhibit melanogenesis by regulating MITF. These agents have all been used to develop anti-melanogenic agents for the treatment of hyperpigmentation disorders. Several studies have also identified plant extracts that have pro-melanogenic response, including the citrus flavonoid naringenin [13], kavalactones [14], coumarin [15], and rosmarinic acid [16]. Naringenin upregulates MITF and tyrosinase through wnt/β-catenin pathway. Rosmarinic acid promotes expression of tyrosinase by activating PKA/CREB pathway. They have been suggested as photo-protecting and pro-melanogenic agents.

Therefore, finding a natural product that is capable of regulating melanin synthesis could contribute to treating melanin-dependent diseases.

*Zingiber cassumunar* Roxb. (Zingiberaceae) is a tropical ginger that is widely distributed in Southeast Asia [17] and has been used as a traditional herbal medicine for gastrointestinal distress and motion sickness [18]. In addition, two main constituents of *Z*. *cassumunar*, phenylbutenoids [19–22] and curcuminoids [23], have been reported to exhibit anti-inflammatory [24,25], anti-tumor [21,22] and antioxidant [23] activities. The inflammatory response is believed to be closely related to melanin synthesis [26,27], and Albert et al. have reported that inflammation of the uveal tract is associated with vitiligo [28]. Therefore, the inflammatory response appears to be a cause of hyperpigmentation diseases in human skin. Despite its effects on the inflammatory response, however, little is known about the effects of *Z*. *cassumunar* on melanogenesis. Therefore, we herein investigated the effects of *Z*. *cassumunar* on melanogenesis.

## Materials and Methods

### Materials and Antibodies

The polyclonal antibody against tyrosinase and the monoclonal antibodies against phospho-ERK, ERK and β-actin were purchased from Santa Cruz (Santa Cruz, CA, USA). The polyclonal antibodies against phospho-p38, p38 were purchased from Cell Signaling (Danvers, MA, USA) and the polyclonal antibody against MITF was purchased from Proteintech (Chicago, IL, USA). The monoclonal antibody against USF1 was purchased from AbCam (Cambridge, MA, USA). The α-MSH and L-DOPA were purchased from Sigma (St. Louis, MO, USA). PD98059 and SB239063 were obtained from Calbiochem (Darmstadt, Germany). U0126 was purchased from Promega (Madison, WI, USA) and SB203580 was purchased from InvivoGen (San Diego, CA, USA). (*E*)-4-(3,4-Dimethoxyphenyl)but-3-en-1-ol (DMPB), (*E*)-4-(3,4-dimethoxyphenyl)but-1,3-diene (DMPBD) and (*E*)-4-(3,4-dimethoxyphenyl)but-3-en-1-yl acetate (DMPBA) were isolated from the chloroform extract of *Z*. *cassumunar* (500 g), as previously described [20].

### Cell culture and transfection

Mouse melanoma cell line B16F10 cells were obtained from ATCC and cultured in Dulbecco’s modified Eagle’s medium (DMEM; WelGene, Daegu, Korea) supplemented with 10% fetal bovine serum (FBS) with gentamicin (50 μg/ml, Sigma) at 37°C in a humidified 5% CO_2_ atmosphere. Primary human epidermal melanocytes were purchased from Lonza (Basel, Switzerland) and maintained in Melanocyte Growth Medium-4 (Lonza), supplemented with 5% FBS, recombinant human-fibroblast growth factor B, rh-insulin, gentamicin sulfate amphotericin-B, calcium chloride, phorbol 12-myristate 13-acetate, bovine pituitary extract and hydrocortisone, at 37°C in a humidified 5% CO_2_ atmosphere. Transient transfections of HEK293T cells were carried out using the Vivamagic reagent (Vivagen, Gyeonggi-Do, Korea). Transient transfections of siRNAs were carried out using the Lipofectamine 2000 reagent purchased from Invitrogen (Carlsbad, CA, USA).

### RNA extraction and reverse transcription polymerase chain reaction (RT-PCR)

Total RNA was extracted from cells and reverse transcribed, and aliquots of the resulting cDNA were amplified using the following primers: mouse tyrosinase (forward) 5'-CGAGCCTGTGCCTCCTCTAA-3' and (reverse) 5'-CCAGGACTCACGGTCATCCA-3'; mouse MITF (forward) 5’-GGAACAGCAACGAGCTAAGG-3’ and (reverse) 5’- TGATGATCCGATTCACCAGA-3’; and β-actin, (forward) 5'-TGGAATCCTGTGGCATCCATGAAA-3' and (reverse) 5'-TAAAACGCAGCTCAGTAACAGTCCG-3'. After an initial denaturation at 94°C for 5 minutes, samples were subjected to 30 cycles of denaturation at 94°C for 30 seconds, annealing at 52°C for 30 seconds, and extension at 72°C for 60 seconds.

### RNA interference

siRNA mediated down regulation of MITF and USF1 was achieved with the MITF specific sequence 5'-GGUGAAUCGGAUCAUCAAG-d(TT)-3' and 5'-CUUGAUGAUCCGAUUCACC-d(TT)-3' and with the USF1 specific sequence 5'-UGGAAGAUCUCAAGAACAA-d(TT)-3' and 5'-UUGUUCUUGAGAUCUUCCA-d(TT)-3'. Scrambled siRNA (siGENOMEnontargetingsiRNA 2) were purchased from Dharmacon (Chicago, IL, USA) and used as a control.

### Western blot

The cultures were washed twice with PBS and the cells were lysed in lysis buffer (50 mM Tris, pH 8.0, 150 mM NaCl, 1% Nonidet P-40, 10 mM NaF, and 2 mM Na_3_VO_4_) containing a protease inhibitor mixture [1 μg/ml aprotinin, 1 μg/ml antipain, 5 μg/ml leupeptin, 1 μg/ml pepstatin A, and 20 μg/ml phenylmethylsulfonyl fluoride (PMSF)]. The total cell lysates were clarified by centrifugation at 13,000 Xg for 15 min at 4°C, denatured with SDS sample buffer, boiled, and analyzed by SDS-PAGE. Nuclear extract was isolated using Nuclear extraction kit (Abcam) and lysates were denatured with SDS sample. The resolved proteins were transferred to polyvinylidene difluoride membranes (Millipore; Billerica, MA, USA), probed with the appropriate antibodies, and detected by ECL (AbClon; Seoul, Korea).

### Quantification of melanin

Melanin contents were measured as described in a previous study [29]. Cells were washed twice with PBS, detached by incubation with trypsin/EDTA, and collected by centrifugation at 1000 Xg for 3 minutes. Thereafter, 5 X10^5^ cells were solubilized in 100 μl of 1 N NaOH-10% DMSO at 80°C for 2 hr. The dissolved melanin was assessed by absorbance at 405 nm, and the melanin content was determined using a standard curve generated with synthetic melanin (Sigma).

### Tyrosinase activity assays

Active tyrosinase was analyzed as described in a previous study [30]. Cells were lysed in 50 mM sodium phosphate buffer (pH 6.8) containing 1% Triton X-100, 1 μM PMSF, 1 μg/ml aprotinin, and 10 μg/ml leupeptin. The lysates were clarified by centrifugation at 13,000 Xg for 15 min at 4°C. Clarified lysates were reacted with 5mM L-DOPA at 37°C for 2 hr, and tyrosinase activity was determined by measuring the absorbance at 470 nm. For analyzing intracellular tyrosinase activity, cells were plated to coverslips in 12-well plates, fixed with 4% paraformaldehyde for 20 min, washed with PBS, and incubated in sodium phosphate buffer with 10 mM L-DOPA for 3 hr at 37°C. The cells were then washed with PBS and the coverslips were mounted on glass slides.

### Immunofluorescence analysis

B16F10 were plated to 12-well plates containing coverslips, and treated with DMPB (30 μM) for 3 hr. Cells were fixed with 3.5% paraformaldehyde and permeablized with 0.5% triton X-100 in PBS. The cells were then washed with PBS, blocked with 0.5% BSA and incubated overnight with the anti-USF1 antibody at 4°C. After a further wash with PBS, the cells were incubated with Texasred conjugated goat anti-rabbit antibody (Invitrogen, Carlsbad, CA, USA) for 1 hr at 25°C. The coverslips were mounted on glass slides with mounting solution containing 4',6-diamidino-2-phenylindole (DAPI), and the results were observed by fluorescence microscopy (Carl Zeiss, Oberkochen, Germany).

### Cell proliferation assay

Cell proliferation was measured using the MTT [3-(4,5-dimethythiazol-2-yl) 2,5-diphenyltetrazolium bromide] assay. In brief, B16F10 cells were harvested with 0.05% trypsin/EDTA and seeded to 96-well plates at 5X10^3^ cells/well. After incubation, medium containing 0.5 mg/ml MTT (100 μl; Sigma) was added to each well, and the cells were incubated for 1 hr. The medium was then removed and 100 μl of acidic isopropanol (90% isopropanol, 0.5% SDS, 25 mM NaCl) was added to each well. The mean concentration of absorbance at 570 nm in each sample set was measured using a 96-well microtiter plate reader (Dynatech; Chantilly, VA, USA).

### Guinea pig model experiments

This study was performed in compliance with the Principles of Laboratory Animal Care and was approved by the Institutional Animal Care and Use Committee (IACUC) of the Asan Institute for Life Sciences, Asan Medical Center (Seoul, Korea). Brownish Kwl:A1 guinea pigs were purchased from Central Lab Animal (Seoul, Korea), anesthetized weekly with a mixture (1:4) of xylazine (Rompun; Bayer Korea, Korea) and Zoletil (Zoletil 50; Virbac, France) given intramuscularly, and shaved. The dorsal skin was separated into three areas (1 cm x 1 cm each), and 100 μM of DMPB, 350 μM of DMPB, or DMSO alone (50 μl each) was topically applied to the center of the allocated area 12 times over 3 weeks. On day 35, skin specimens were obtained by 5-mm punch biopsies, embedded in Tissue-Tek OCT compound (Sakura Fine Technical; Tokyo, Japan), and quickly frozen. The frozen specimens were cut to 10-μm thickness and fixed in ice cold acetone for 10 min. Melanin pigment was visualized with standard Fontana—Masson staining. Image analysis was performed on a representative area of three randomly selected fields using the ImageJ program (http://rsb.info.gov/ij/). For melanocyte counting, sections were stained with Hematoxylin and Eosin, and random fields were examined under a microscope (x400). Dermatologists counted the melanocytes within four different fields from a 0.5-mm length of epidermis. All animals were sacrificed with CO_2_ inhalation after study.

### Statistical analysis

Data are presented as the means from three independent experiments. Statistical analyses were performed using the unpaired Student’s t test. A p-value less than 0.01 or 0.05 was considered statistically significant.

## Results

### (E)-4-(3,4-Dimethoxyphenyl)but-3-en-1-ol from *Z*. *cassumunar* enhances melanin synthesis

**Fig 1:**
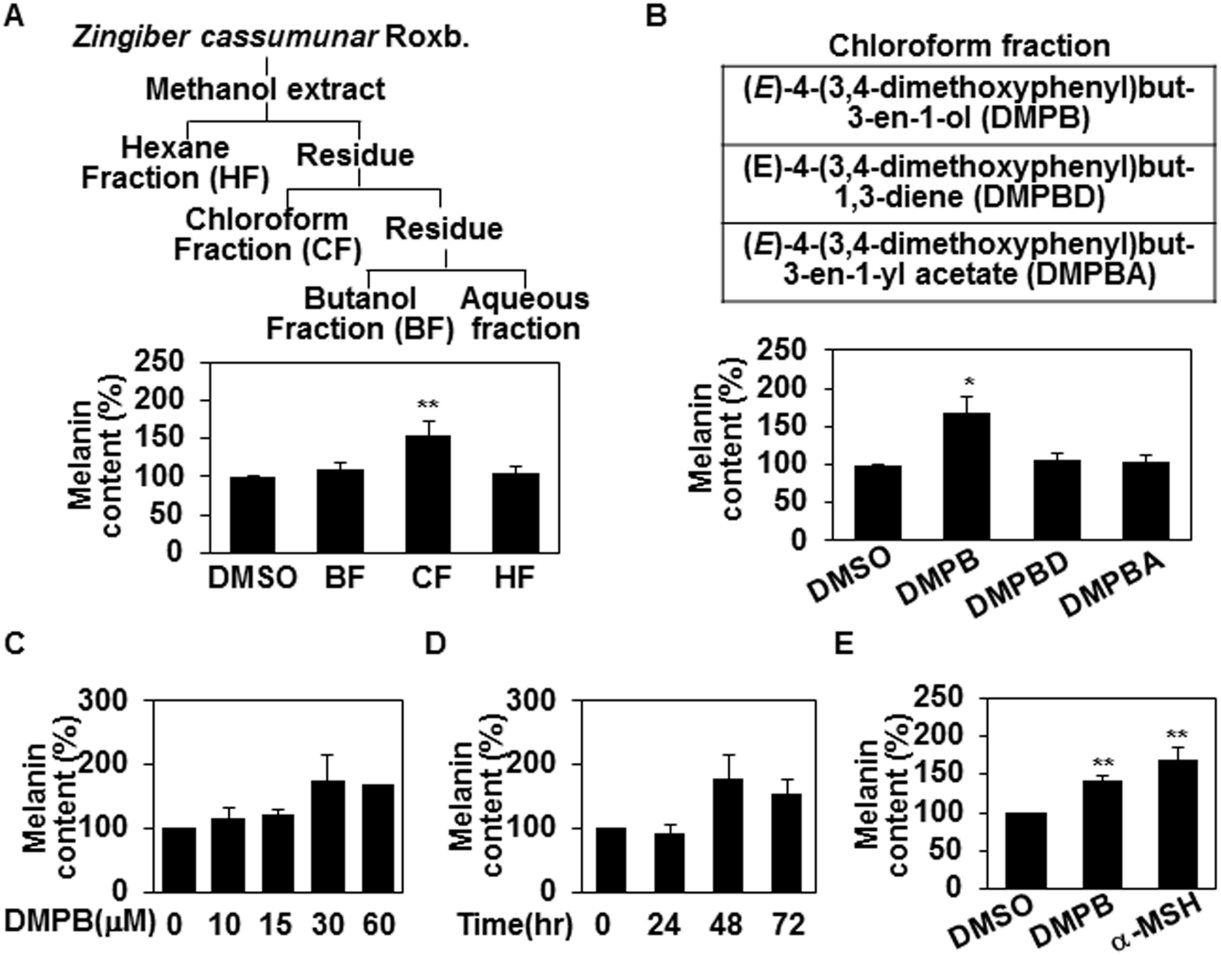
(E)-4-(3,4-Dimethoxyphenyl)but-3-en-1-ol from *Z*. *cassumunar* enhances melanin synthesis. (A) The methanol extract of *Z*. *cassumunar* was partitioned with hexanes, chloroform, and butanol (*top panel*). B16F10 cells were treated with three fractions of *Z*. *cassumunar* (BF: Butanol fraction, CF: Chloroform fraction, HF: Hexane fraction; 20 μg/ml, 48hr). The melanin contents were analyzed by measuring the absorbance at 405 nm (*bottom panel*). DMSO was used as a control. The mean percentages of melanin content are shown. **, p < 0.01 versus DMSO treated cells. (B) B16F10 cells were treated with the indicated compounds extracted from *Z*. *cassumunar* (30 μM each) for 48 hr, and the melanin contents were determined. *, p < 0.05 versus DMSO treated cells. (C,D) B16F10 cells were treated with either various concentrations of DMPB for 48 hr (C) or with 30 μM of DMPB for the indicated times (D), and the mean percentages of melanin content are shown. (E) B16F10 cells were treated with of 30 μM of DMPB or 1 μM of α-MSH for 48 hr. The mean percentages of melanin content are shown. **, p < 0.01 versus DMSO treated cells.

The methanol extract of *Z*. *cassumunar* was partitioned with hexanes, chloroform, and butanol, subsequently, as described previously [20] and then compared the melanin contents of B16F10 mouse melanoma cells in the presence or absence of these extracts (20 μg/ml) for 48 hr. We found that chloroform extract, but not hexane or butanol extracts, enhanced melanin synthesis in B16F10 mouse melanoma cells (Fig 1A). From the chloroform fraction, we isolated three compounds [20]: (*E*)-4-(3,4-dimethoxyphenyl)but-3-en-1-ol (DMPB), (*E*)-4-(3,4-dimethoxyphenyl)but-1,3-diene (DMPBD) and (*E*)-4-(3,4-dimethoxyphenyl)but-3-en-1-yl acetate (DMPBA). As shown in Fig 1B, we repeated the above assay to determine which compound affected melanogenesis, and found that only DMPB increased melanin synthesis in B16F10 cells (Fig 1B), showing a dose-dependent effect that was maximum among cells treated with 30 μM (Fig 1C) for 48 hr (Fig 1D). These effects were found to be comparable (though slightly lower) than those of α-melanocyte stimulating hormone (α-MSH) (Fig 1E). Together, these data suggest that DMPB extracted from *Z*. *cassumunar* enhances melanin synthesis in B16F10 melanoma cells.

### DMPB enhances tyrosinase expression but not tyrosinase activity

**Fig 2:**
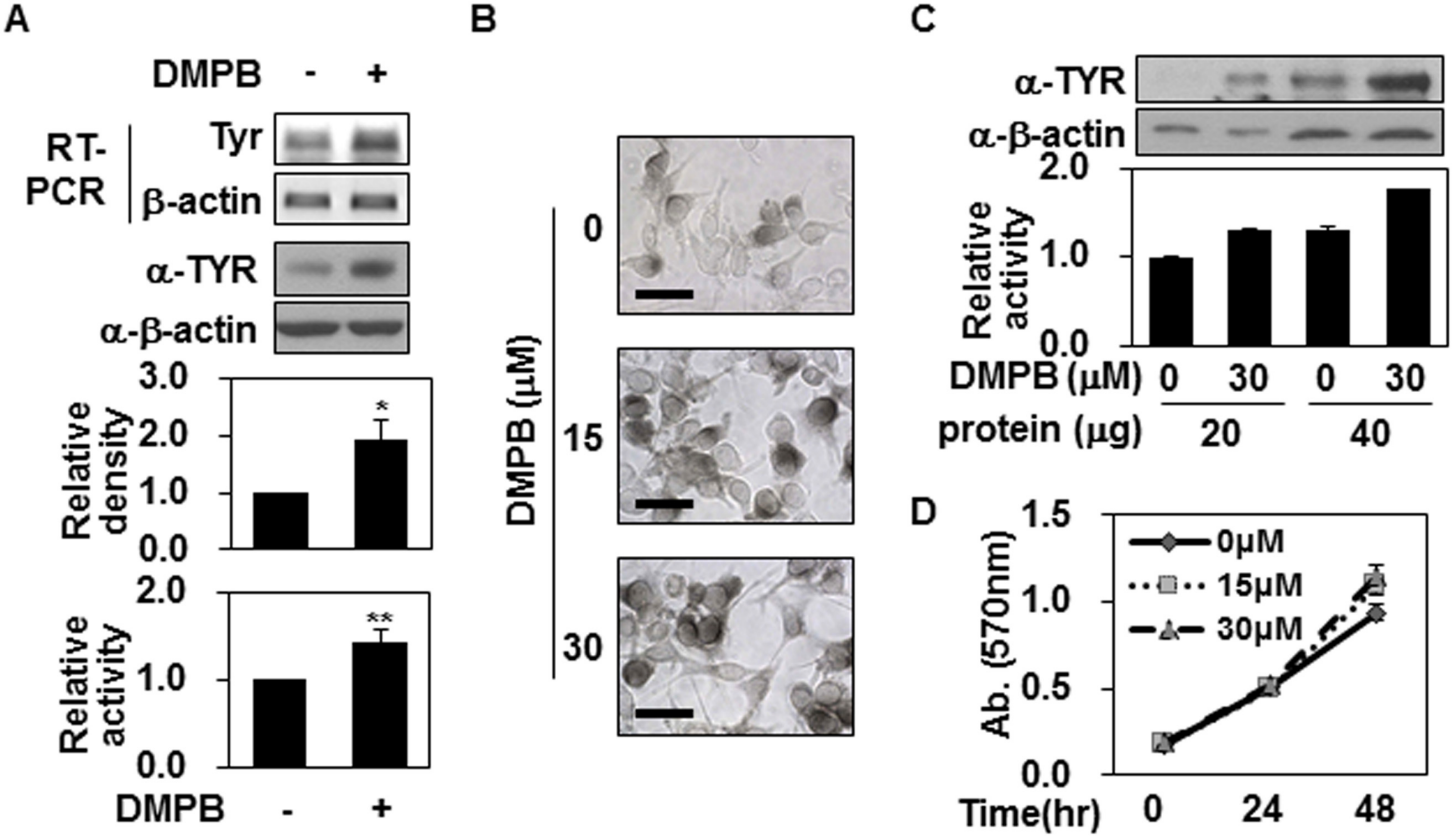
DMPB increases tyrosinase expression but not tyrosinase activity. (A) B16F10 cells were treated with 30 μM of DMPB for 48 hr, and mRNA level of tyrosinase was analyzed by RT-PCR (*top panel*). Total cell lysate was extracted and tyrosinase levels were measured by Western blot analysis. The relative density of tyrosinase(TYR) was quantitated using Image Studio software (*middle panel*). The mean percentages of tyrosinase density ± SD are shown *, p < 0.05 versus DMSO treated cells. DMPB-treated B16F10 cells (30 μM, 48 hr) were lysed. Cell lysates (100 μg) were reacted with L-DOPA at 37°C for 2 hr, and tyrosinase activity was determined at 470 nm (*bottom panel*). The mean percentages of tyrosinase activity ± SD are shown **, p < 0.01 versus DMSO treated cells. (B) DMPB-treated B16F10 cells (48 hr) were reacted with L-DOPA at 37°C for 30 min. Bright-field microscopic images are shown. Scale bars = 50 μm. (C) Cell lysates (20 μg and 40 μg) from B16F10 cells treated with the indicated concentrations of DMPB were subjected to Western blot analysis using an anti-tyrosinase antibody (*top panel*) or reacted with L-DOPA at 37°C for 2 hr to determine tyrosinase activity (*bottom panel*). The mean percentages of tyrosinase activity ± SD are shown. (D) B16F10 cells were incubated with various concentrations of DMPB for the indicated time periods, and cell viability was determined by MTT assay. Percentage values were compared between treated and untreated (control). Data are expressed as mean ± SD for three independent experiments.

Since DMPB increased the levels of melanin in our system, we next investigated whether it could affect the expression of tyrosinase, which plays a critical role in melanogenesis. As shown in Fig 2A, Western blotting and RT-PCR analyses revealed that the expression levels of tyrosinase were significantly up-regulated in B16F10 cells treated with 30 μM of DMPB for 48 hr. Similarly, tyrosinase activity showed that total tyrosinase activity increased in response to DMPB treatment (Fig 2A). L-DOPA staining showed that the level of intracellular tyrosinase activity was increased in DMPB-treated B16F10 cells (Fig 2B), confirming the abovementioned increase in total tyrosinase activity among DMPB-treated cells. However, when we adjusted the amount of tyrosinase in the reaction mixture, the tyrosinase activity was comparable in B16F10 cells with and without DMPB treatment (Fig 2C). This suggests that the increase of tyrosinase activity in DMPB-treated B16F10 cells was due to increased tyrosinase levels rather than increased tyrosinase activity. Since increased cell numbers might affect the total tyrosinase activity, we used a colorimetric assay to investigate whether DMPB affected the proliferation of B16F10 cells, but found that cell number increased similarly over time in the presence or absence of DMPB (Fig 2D), suggesting that this agent does not affect the proliferation of B16F10 cells. Together, these findings support our contention that DMPB increases melanin synthesis by up-regulating tyrosinase expression.

### MAP kinases are involved in DMPB-mediated melanogenic control

**Fig 3:**
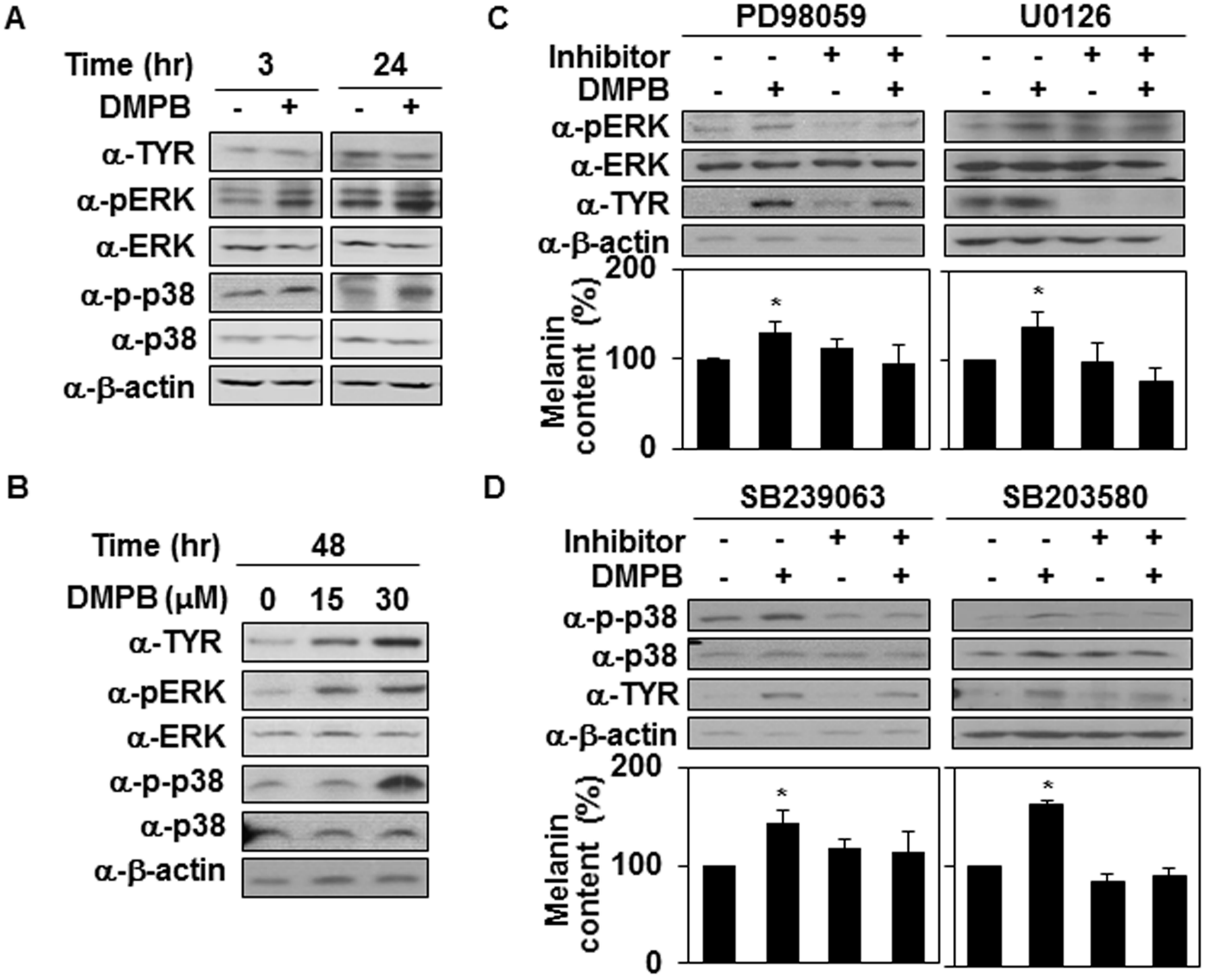
MAP kinases are involved in DMPB-mediated melanogenic control. (A) B16F10 cells were treated with 30 μM of DMPB for the indicated time periods, and the phosphorylation of p38 and ERK and levels of tyrosinase were analyzed by Western blot analysis. (B) B16F10 cells were treated with the indicated amounts of DMPB for 48 hr, and the phosphorylation of p38 and ERK and levels of tyrosinase were analyzed by Western blot analysis. (C) B16F10 cells were preincubated with (+) or without (-) the inhibitor (1 μM of PD98059, 10 μM of U0126) for 1 hr, then treated with 30 μM of DMPB for 48 hr, and Western blot analysis was performed with the indicated antibodies (*top panel*). The melanin contents were analyzed by measuring the absorbance at 405 nm (*bottom panel*). The mean percentages of melanin content are shown *, p < 0.05 versus DMSO treated cells. (D) B16F10 cells were preincubated with (+) or without (-) p38 inhibitors (5 μM of SB239063 for 30 min, 10 μM of SB203580 for 1 hr) and treated with 30 μM of DMPB for 48 hr, and Western blot analysis was performed with the indicated antibodies (*top panel*). The melanin contents were analyzed by measuring the absorbance at 405 nm (*bottom panel*). The mean percentages of melanin content are shown *, p < 0.05 versus DMSO treated cells.

The mitogen-activated protein kinase (MAPK) signaling pathway is known to be involved in regulating melanin synthesis by modulating the expression of tyrosinase. UV irradiation and α-MSH have been shown to activate p38 MAPK, subsequently up-regulating the expression of tyrosinase [4,12,31]. In addition, ERK activation phosphorylates cAMP response element binding protein (CREB), which binds to the CRE consensus motif in the MITF promoter to up-regulate MITF gene expression [32]. Therefore, we next investigated whether DMPB affects the MAPK signaling cascade. In particular, we compared the activity levels of ERK and p38 using Western blotting with phospho-specific antibodies. At 3 hr after treatment, compared with untreated control cells, DMPB-treated cells increased the activity of ERK and p38 kinase (Fig 3A) but not Jnk (data not shown), suggesting that DMPB-mediated melanogenesis is closely related to increases in MAPK activity. Consistently, the activity of ERK and p38 kinase remained increased for 48 hr (Fig 3B). When B16F10 cells were pretreated with PD98059 and U0126 (a specific inhibitor of MEK), we observed decreases in various DMPB-induced effects, including ERK phosphorylation, tyrosinase expression and melanin synthesis (Fig 3C). Similarly, SB239063 and SB203580 (a specific inhibitor of p38) alleviated DMPB-induced p38 phosphorylation, tyrosinase expression and melanin synthesis in B16F10 cells (Fig 3D). These findings indicate that both ERK and p38 seem to be involved in DMPB-mediated melanogenic control.

### DMPB enhances melanogenisis is an upstream stimulating factor-1-dependent fashion

**Fig 4:**
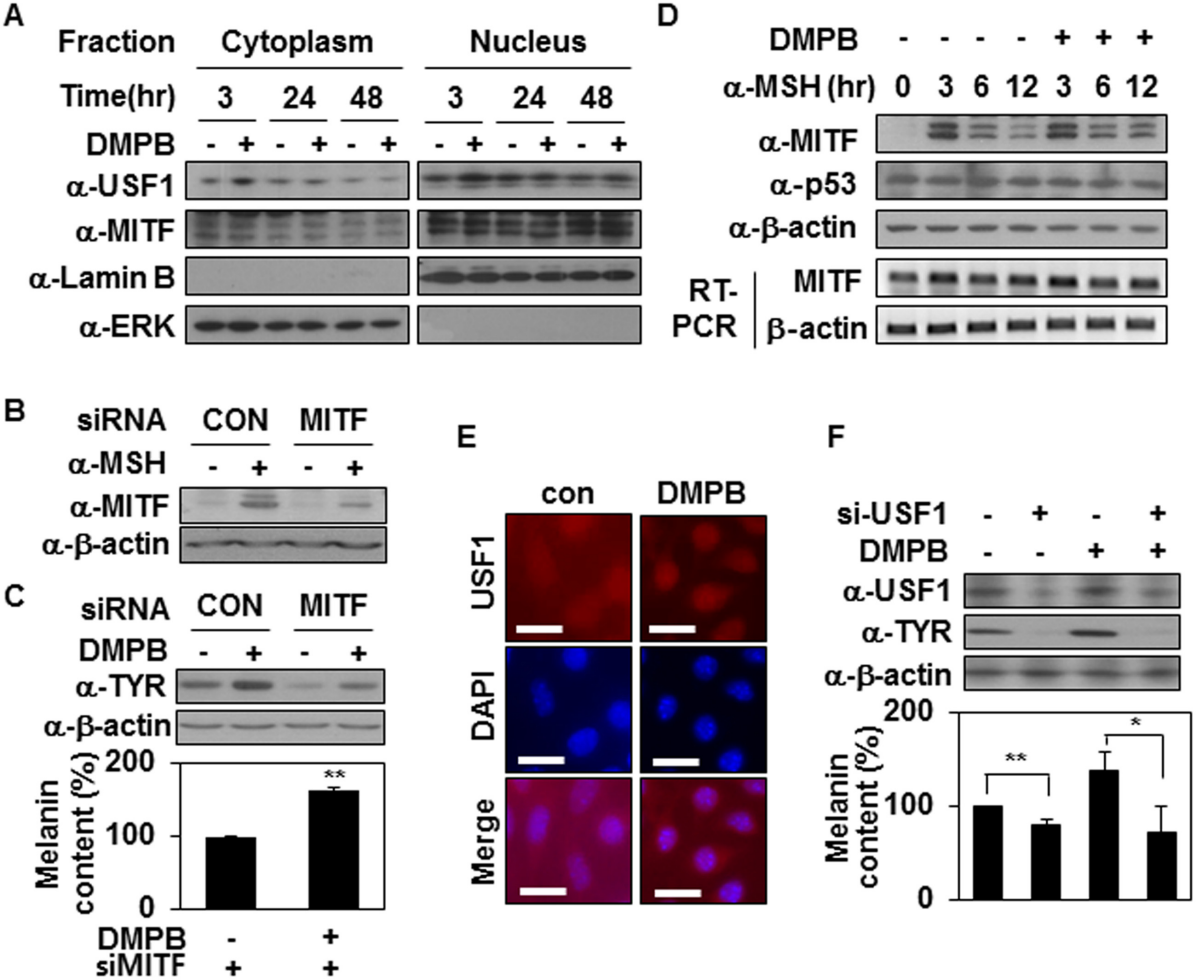
DMPB stimulates melanin synthesis by activating USF1. (A) B16F10 cells were treated with 30 μM of DMPB for the indicated time periods. Cytoplasmic and nuclear fractions were isolated and analyzed by Western blotting. (B) B16F10 cells were transfected with si-CON or si-MITF for 48 hr. MITF protein levels were analyzed by Western blotting in the absence or presence of 1 μM α-MSH for 3 hr. (C) Tyrosinase protein levels were analyzed by Western blotting in the absence or presence of 30 μM DMPB for 48 hr (*top panel*) and the melanin content was measured by absorbance at 405 nm (*bottom panel*). The mean percentages of melanin content are shown. **, p < 0.01 versus DMSO treated cells. (D) B16F10 cells were treated with 30 μM of DMPB for the indicated time periods in the presence of 1 μM α-MSH. MITF and p53 protein levels were analyzed by Western blotting and MITF mRNA level was analyzed by RT-PCR. (E) B16F10 cells were treated with DMPB for 3 hr, and Immunofluorescence analysis was performed with anti-USF1 antibody. Scale bar = 20 μm (F) B16F10 cells were transfected with si-con or si-USF1 and treated with DMPB (30 μM) for 48 hr. Indicated protein levels were analyzed by Western blotting (*top panel*) and the melanin content was measured by absorbance at 405 nm (*bottom panel*).**, p<0.01; *, p < 0.05 versus DMSO treated cells.

It has been reported that tyrosinase gene transcription is regulated by several transcription factors including MITF, p53 and USF1 [4,33,34]. MITF is a basic helix—loop—helix leucine zipper (bHLH-LZ) transcription factor that binds the tyrosinase gene promoter region to activate tyrosinase gene expression [3]. Therefore, we speculated that MITF might be involved in DMPB-mediated melanogenic regulation. We used Western blotting to analyze the levels of MITF in B16F10 cells treated with DMPB. However, DMPB did not affect MITF expression (Data not shown) and nuclear translocation of MITF (Fig 4A), suggesting that DMPB does not affect MITF expression. To further investigate the potential involvement of MITF in the regulation of DMPB-mediated melanin synthesis, we used unique siRNA sequences targeted against MITF to knock down the expression levels of MITF. B16F10 transfected with the siRNA constructs showed decreased expression of MITF induced by α-MSH treatment (Fig 4B), supporting that siRNA mediated knockdown of MITF expression. Interestingly, siRNA-mediated knockdown of MITF expression reduced basal tyrosinase expression but did not suppress DMPB-mediated tyrosinase expression (Fig 4C, top panel) or melanin synthesis (Fig 4C, bottom panel). This suggests that DMPB regulates tyrosinase expression independently of MITF.

As regulation of MITF stability is also a key feature of melanogenic signaling [35], we examined the effect of DMPB on the degradation of MITF. B16F10 cells were treated with α-MSH, a well-known inducer of MITF, and then the subsequent degradation of MITF was analyzed over time. As shown in Fig 4D, the levels of MITF were dramatically enhanced in B16F10 cells at 3 hr after treatment with α-MSH, but decreased thereafter, returning to the basal level by 6 hr post-treatment. B16F10 cells treated with DMPB showed the same degradation pattern following α-MSH treatment, indicating that DMPB is not involved in regulating MITF. Similarly, the levels of p53 were not affected in response to DMPB (Fig 4D). Interestingly, however, total protein levels and nuclear levels of USF1 were increased in response to DMPB at 3 hr (Fig 4A). In addition, DMPB enhanced nuclear localization of USF1 (Fig 4E), suggesting that DMPB regulates tyrosinase expression through regulating levels of USF1. Expectedly, DMPB-mediated melanin synthesis was abolished when USF1 expression was reduced by siRNA targeting USF1 (Fig 4F). All these data suggest that DMPB regulates tyrosinase expression and melanin synthesis through regulating levels of USF1.

### DMPB promotes melanin synthesis in human melanocytes

**Fig 5:**
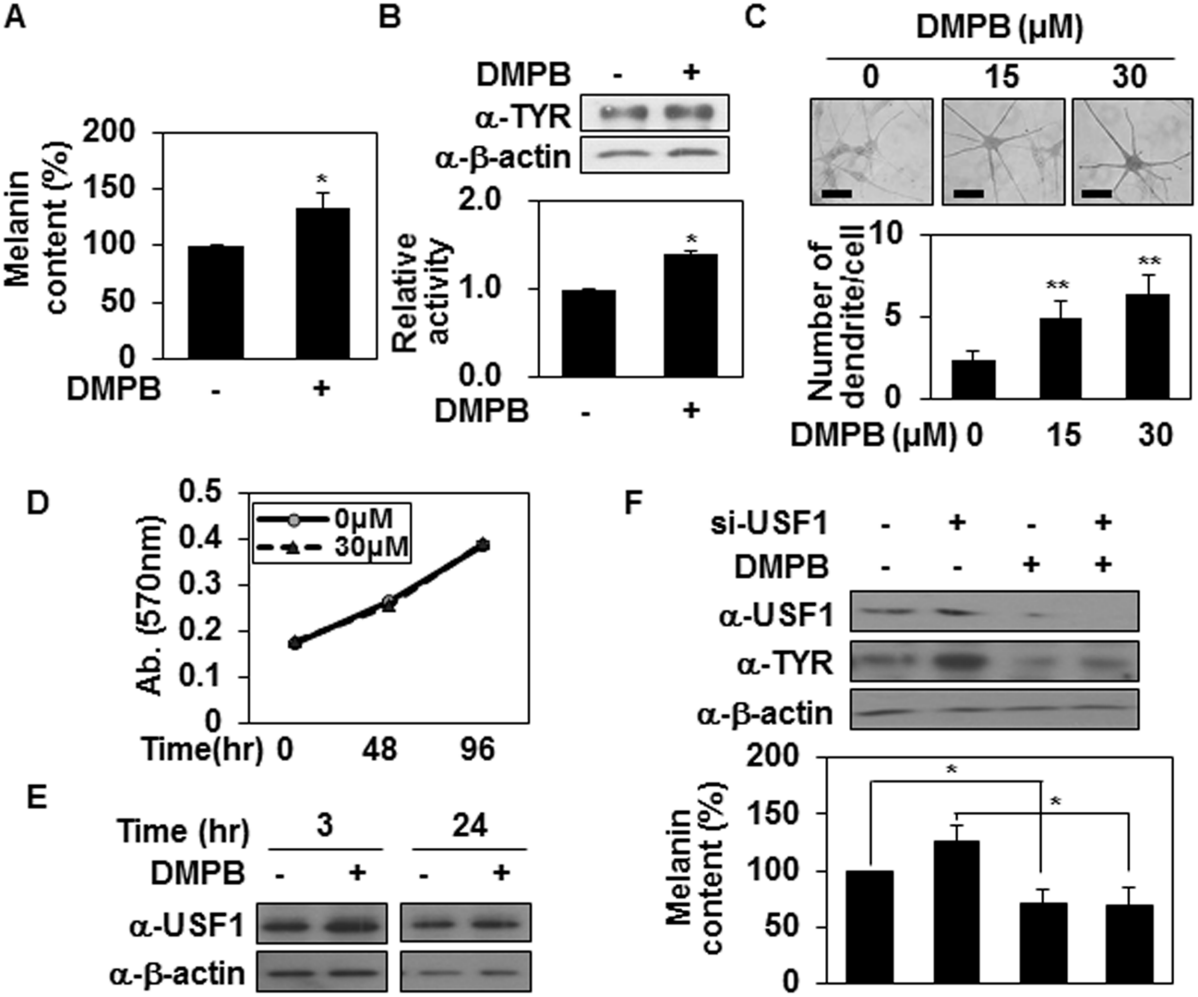
DMPB promotes melanin synthesis in human melanocytes. (A, B) Human melanocytes were treated with 30 μM of DMPB. After 48 hr melanin contents were analyzed by measuring the absorbance at 405 nm (A). The mean percentages of melanin content are shown *, p < 0.05 versus DMSO treated cells. Tyrosinase expression levels were analyzed by Western blot analysis (B; *top panel*). Cell lysates (100 μg) were reacted with L-DOPA at 37°C for 2 hr, and tyrosinase activity was determined at 470 nm (B; *bottom panel*). The mean percentages of tyrosinase activity ± SD are shown. *, p < 0.05 versus DMSO treated cells. (C) Human melanocytes were plated on 12-well plates, treated with the indicated concentrations of DMPB for 48 hr, and reacted with L-DOPA at 37°C for 30 min. Bright-field microscopic images are shown (*top panel*). Scale bars = 50 μm. The mean percentages of the number of dendrite/cell ± SD are shown (*bottom panel*). **, p < 0.01 versus DMSO treated cells. (D) Primary melanocytes were incubated with DMPB (30 μM) in a 96-well plate for the indicated periods, and cell viability was determined by MTT-based spectrophotometric assay. Percentage values were compared between treated and untreated (control) cells. The data are expressed as mean ± SD from three independent experiments. (E) Primary melanocytes were treated with 30 μM of DMPB for the indicated periods. USF1 protein levels were analyzed by Western blotting. (F) Primary melanocytes were transfected with USF1 targeting siRNA and treated with DMPB (30 μM). After 48 hr, tyrosinase and USF1 expression levels were analyzed by Western blot analysis (*top panel*). Cells were harvested and melanin contents were analyzed by measuring the absorbance at 405 nm (*bottom panel*). The mean percentages or melanin content are shown *, p < 0.05.

We next investigated whether DMPB could regulate melanin synthesis in human melanocytes (Fig 5). Consistent with our findings in mouse melanoma cells, DMPB (30 μM) treatment of human melanocytes triggered increases in melanin synthesis (Fig 5A), tyrosinase expression (Fig 5B, top panel), total tyrosinase activity, (Fig 5B, bottom panel), and intracellular tyrosinase activity together with increased dendrite formation (Fig 5C), whereas DMPB (30 μM) did not affect on the proliferation of human melanocytes (Fig 5D). In addition, DMPB induced levels of USF1 (Fig 5E) and DMPB-mediated melanin synthesis was dependent on USF1 like melanoma cells (Fig 5F). Taken together, our results indicate that DMPB affects melanogenesis in human primary melanocytes.

### DMPB enhances hyperpigmentation in brown guinea pigs *in vivo*

**Fig 6:**
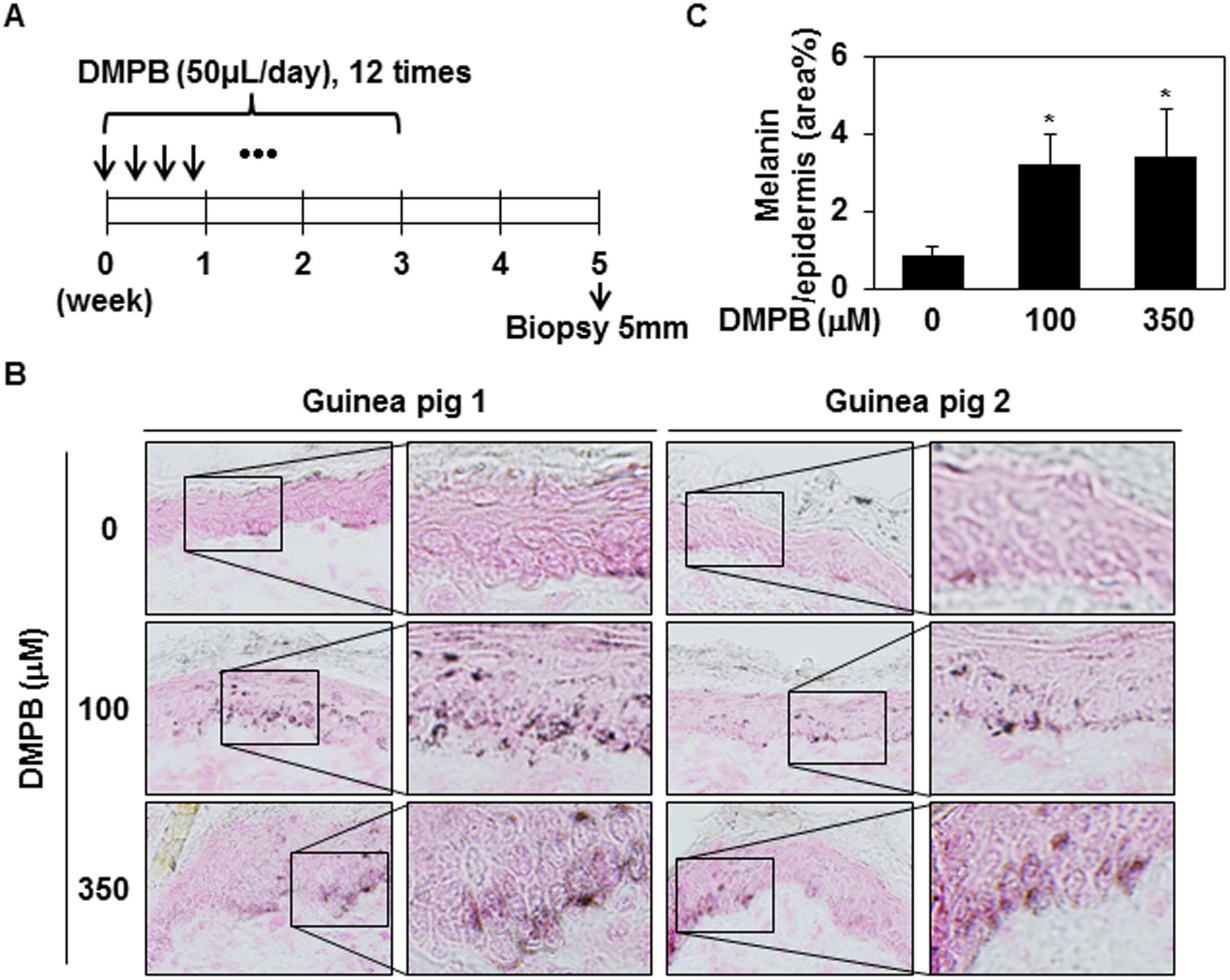
DMPB enhances hyperpigmentation in brown guinea pigs. (A) The dorsal skins of guinea pigs were topically treated with DMSO (control), 100 μM DMPB, or 350 μM DMPB (50 μl each) 12 times in 3 weeks. On day 35 (after the first treatment), skin specimens were obtained by a 5-mm punch biopsy. (B) Frozen skin specimens were cut at 10- μM thickness, and melanin pigment was visualized by Fontana-Masson staining. Original magnification, X200. (C) Bar graph showing the mean percentage of melanin ± SD in the epidermis. Fontana-Masson-stained melanin in three randomly selected fields was measured with the ImageJ program. *, p < 0.05 versus DMSO treated skins.

We next examined the promelanogenic effect of DMPB *in vivo* using guinea pigs. Brownish Kwl:A1 guinea pigs were treated with either 100 μM or 350 μM of DMPB, or DMSO (n = 2 per group), as shown in Fig 6A. After 38 days, frozen skin specimens were sectioned and subjected to Fontana-Masson staining. As shown in Fig 6B and 6C, enhanced hyperpigmentation was seen in skin samples from DMPB-treated guinea pigs compared with DMSO-treated animals. More specifically, DMPB treatment significantly increased melanin synthesis in the epidermal basal layer, but did not affect the number of melanocytes in the epidermis. Taken together, these results demonstrate that DMPB positively regulates melanin synthesis *in vivo*.

## Discussion

We herein provide the first evidence that DMPB isolated from *Z*. *cassumunar* stimulates melanin synthesis via the ERK and p38 MAPK signaling pathways. DMPB treatment increased melanin synthesis in B16F10 mouse melanoma cells (Figs 1 and 2) and human primary melanocytes (Fig 5). The DMPB-induced melanogenic response in B16F10 cells was comparable to that induced by α-MSH treatment. Furthermore, the promelanogenic effect of DMPB was evident in an *in vivo* guinea pig model (Fig 6). Therefore, our results indicate that DMPB enhances melanin synthesis in melanocyte-derived cells.

To understand the mechanism underlying this DMPB-induced increase in melanin synthesis, we investigated the effect of DMPB on the expression and activity levels of tyrosinase, which mediates the cellular melanin content (Fig 2). DMPB treatment was found to increase tyrosinase at both the protein and mRNA levels, suggesting that DMPB regulates tyrosinase at the transcriptional level.

Members of the MAP kinase family, including ERK, Jnk, and p38 MAPK, play important roles in melanogenesis [36]. Activation of the p38 and ERK MAPK pathways activates MITF, which subsequently induces expression and activation of tyrosinase in zebrafish embryos and B16F10 cells [37]. ERK stimulates MITF transcription via the transcription factor, BRN2 [38]. On the other hand, phosphorylated ERK has also been shown to induce the phosphorylation and subsequent degradation of MITF phosphorylation, thereby reducing melanin synthesis [39,40]. A number of studies have shown that p38 MAPK is involved in melanin synthesis including stress-induced melanogenesis. Both UV irradiation and hydrogen peroxide have been shown to activate p38 MAPK, which plays an important role in melanogenesis by stimulating USF1 and upregulating tyrosinase expression [41]. LPS stimulates expression of both MITF and tyrosinase through p38 MAPK [42]. Mansky et al. showed that activation of the p38 MAPK pathway results in the phosphorylation of MITF on serine 307 and the subsequent upregulation of MITF target genes in osteoclasts [43]. In parallel with increased melanin synthesis, we found that the phosphorylation levels of ERK and p38, but not Jnk, were significantly enhanced after DMPB treatment, suggesting that the DMPB-mediated melanogenic effects may occur via MAPK-mediated pathways (Fig 3A and 3B). Consistent with this notion, the MAPK-specific inhibitors, PD98059, U0126, SB239063 and SB203580, reduced the DMPB-mediated increase of melanin synthesis (Fig 3C and 3D). However, DMPB did not affect the level of MITF (Fig 4A and 4D). Therefore, DMPB seems to regulate melanin synthesis by altering tyrosinase expression in an MITF-independent manner.

Another bHLH-LZ transcription factor, USF1 is regulated by various signaling pathways, including ERK1/2 and p38 MAPK. In HepG2 cells, activation of ERK mediated by HGF phosphorylates of USF1 [44]. In response to UV stress, activation of p38 MAPK/USF1 appears essential for protecting skin through enhancement of melanogenesis [45]. Both ERK1/2 and p38 MAPKs phosphorylate at Ser153 site of USF1 [46]. USF1 stimulates melanin synthesis as a transcription factor of tyrosinase, MC1R and POMC [4, 45], and maintains genomic stability via its involvement in DNA repair [47]. Therefore, it is highly possible that USF1 plays a role in DMPB-mediated melanogenesis. Our data clearly showed that USF1 regulates DMPB-mediated melanogenesis as a transcription factor of tyrosinase (Fig 4). DMPB enhanced the level of USF1 (Figs 4A and 5E) and stimulated its translocation to the nucleus (Fig 4A and 4E). In addition, siRNA knockdown of USF1, but not MITF (Fig 4C), led to significant inhibition of tyrosinase-mediated melanogenesis by DMPB (Figs 4F and 5F). Based on these results, we propose that DMPB promotes melanin synthesis through increasing USF1-mediated tyrosinase expression.

In this study, we found new pro-melanogenic agent, DMPB. This DMPB clearly enhances melanin synthesis by increasing tyrosinase expression, as shown in cultured melanocyte-derived cells *in vitro* and in guinea pig skin *in vivo*. These results provide early evidence that DMPB could act as a pigmenting agent *in vivo*, and might be useful for treating hypopigmentation-related disorders. Further studies are needed to directly examine the effect of DMPB on skin pigmentation under physiologically relevant conditions.

## Abbreviations

DMPB: (E)-4-(3,4-dimethoyphenyl)but-3-en-1-ol
USF1: Upstream stimulating factor-1
MITF: microphethalmia transcription factor
ERK: extracellular signal-regulated kinase
TYR: tyrosinase
L-DOPA: L-3,4-dihydroxyphenylalanine
